# Surgical Management of Sacral Bone Tumors: A Retrospective Analysis of Outcomes, Complications, and Survival

**DOI:** 10.3390/diagnostics15070917

**Published:** 2025-04-02

**Authors:** Chiara Cini, Emanuela Asunis, Cristiana Griffoni, Gisberto Evangelisti, Giuseppe Tedesco, Riccardo Ghermandi, Marco Girolami, Valerio Pipola, Silvia Terzi, Giovanni Barbanti Brodano, Stefano Bandiera, Stefano Boriani, Alessandro Gasbarrini

**Affiliations:** 1 Department of Spine Surgery, IRCCS Istituto Ortopedico Rizzoli, 40136 Bologna, Italy; chiara.cini@ior.it (C.C.); e.asunis@santobonopausilipon.it (E.A.); gisberto.evangelisti@ior.it (G.E.); giuseppe.tedesco@ior.it (G.T.); riccardo.ghermandi@ior.it (R.G.); marco.girolami@ior.it (M.G.); valerio.pipola@ior.it (V.P.); silvia.terzi@ior.it (S.T.); giovanni.barbantibrodano@ior.it (G.B.B.); stefano.bandiera@ior.it (S.B.); alessandro.gasbarrini@ior.it (A.G.); 2 Department of Pediatric Orthopedics and Traumatology, A.O.R.N. Santobono Pausilipon, 80129 Napoli, Italy; 3 Spine Unit, Hospital Universitario Fundación Jiménez Díaz, 28040 Madrid, Spain; 4 Alma Mater Studiorum University of Bologna, 40126 Bologna, Italy; sb@stefanoboriani.eu; 5 Department of Biomedical and Neuromotor Sciences, Alma Mater Studiorum University of Bologna, 40126 Bologna, Italy

**Keywords:** primary malignant tumors, sacral localization, sacrectomy

## Abstract

**Background**: Primary malignant bone tumors are exceedingly rare, with an incidence of 0.5 to 1 per million, and sacral localization is even more uncommon, representing only 1–3.5% of these tumors. These malignancies are often diagnosed late due to their asymptomatic nature until they present as large, advanced intrapelvic tumors. Management is complicated by the need for precise surgical intervention and the consideration of adjuvant therapies based on tumor histology and patient factors. **Methods**: We conducted a single-center, retrospective analysis of patients who underwent complete, partial, or hemisacrectomy for primary malignant bone tumors or recurrent sacral metastases. Excluded were patients with metastatic disease not necessitating sacrectomy. Data collected included demographics, clinical characteristics, tumor types, resection status, adjuvant therapies, recurrence, metastasis, and complications. Surgical approaches were categorized as posterior, anterior, or combined anterior–posterior. The primary outcomes were overall survival and disease-free survival, while the secondary outcomes focused on complication rates and functional outcomes. **Results**: The study included 19 patients (7 females, 12 males) with a mean age of 48.9 years at the time of surgery. Primary malignancies were present in 90% of patients. Surgical approaches varied: 20% underwent double access and 5% anterior access only, and the remainder had posterior approaches. High partial sacrectomy (above S3) was performed in 20%, while low sacrectomy (at or below S3) was performed in 80%. Complete resection with clean margins (R0) was achieved in 65% of cases, while 35% had R1 resections with microscopic tumor remnants. Root resection was necessary in 25% of patients. Local recurrence occurred in 25% of patients, with two requiring reoperation and neurological sacrifice. Distant metastases were observed in 20% of cases. Postoperative complications affected 60% of patients. The most common issues were surgical wound dehiscence with delayed healing (35%) and visceral changes affecting the bowel and urination (25%). No mechanical complications were reported. **Conclusions**: Sacrectomy remains a challenging procedure with substantial morbidity and variability in outcomes. The choice of surgical approach—posterior, anterior, or combined—depends on tumor location and extent. While posterior-only approaches are often preferred for lower sacral lesions, combined approaches may be necessary for more extensive tumors. Survival and disease-free survival rates are influenced by resection margins and the biological behavior of the tumor. Wide-margin resections (R0) are associated with lower local recurrence rates but do not eliminate the risk of distant metastases.

## 1. Introduction

Tumors of the sacrum and related neural, retroperitoneal, and pelvic structures are rare, accounting for 1 to 7% of all spinal tumors. Their diagnosis is frequently delayed, and the neoplasm may be far advanced at the time of presentation [1]. Moreover, solitary metastases in the sacral region are uncommon, as evidenced by the low number of case series [2,3,4]. Regarding sacral metastases, studies have most frequently identified renal cell carcinoma [3,5], lung carcinoma [5], and breast carcinoma [6]. Generally, sacral malignancies are diagnosed incidentally or when they give symptoms present with large and advanced intrapelvic tumors [2,4]. The type of surgical approach, as well as the choice to perform eventual neoadjuvant or postoperative therapy depends on the histology of the tumor, but certainly also on the performance status of the patient. In the present case, tumor biology, eligibility for radiation treatment, and response to chemotherapy, as well as any neurological impairment, guide us toward treatment. It is known that intralesional resection without adequate surgical, and consequently histological, margins can lead to a higher rate of local recurrence as well as eventual spread of disease [5,7,8,9]. In other studies, en bloc resection or wide-margin resection combined with high-dose adjuvant proton therapy has been widely shown to increase the period of disease-free survival in patients with sacral neoplasms that, by their molecular biology, do not respond to conventional chemotherapy and radiotherapy [5,7,8]. Although the advantage of aggressive surgical treatment is clear, in terms of low recurrence rate and distant metastasis, it is clear that, as previously mentioned, the surgery must always be tailored to the patient, location, and type of tumor.

Clinically, partial and distal sacrectomy is usually well tolerated and can be performed without much difficulty in terms of neurological sacrifice. The problem arises with extensive intrapelvic expansion or tumor growth exceeding the S2 segment. In this case, surgery would require a high or total sacrectomy that usually requires the sacrifice of S1/S2 nerve roots with associated sensorimotor dysfunction; moreover, there is the risk of vertical and rotational pelvic instability that often requires spinopelvic fixation to allow effective pain control and enable better ambulatory capacity [3,10,11]. Currently, the scientific landscape describes different surgical resection techniques and different reconstruction options [8,12,13,14]. However, early and late postoperative complication rates continue to be relatively high, which is why their management requires challenging interdisciplinary management [1]. The additional problem is dictated by the low incidence of some tumor entities and their rare localization in the sacrum, which is why data on the surgical resection technique, associated complications, and oncologic outcome are currently limited and in the process of being defined. Our study aims to contribute to this developing scenario.

## 2. Materials and Methods

The data for this study were collected retrospectively from the electronic health records of patients surgically treated from 2016 to 2023 at Istituto Ortopedico Rizzoli. The patients provided informed consent for an observational study (registry) approved by the local ethics committee on 14 December 2016 (protocol number 0022814).

Demographic, clinical, and pathological data, such as sex, age at diagnosis, tumor type, and resection status after surgery, metastasis, local recurrence, and neoadjuvant or adjuvant therapies, were collected and subsequently analyzed. Operative data included level of bone resection, number and level of nerve roots sacrificed, surgical margins, type of soft tissue reconstruction, and method of spinopelvic stabilization. We divided sacral resection according to the localization of the tumor: those used for midline tumors and those used for eccentric lesions involving the sacro-iliac joint (SIJ). The midline group included low, middle, and high sacral amputations, total sacrectomy, and hemicorporectomy defined by the line of resection and by the level of root sacrificed. The lateral group of sacral resections included en bloc excision of the sacroiliac joint and hemisacrectomy. Patients treated with posterior and combined anterior–posterior approaches were included in our study. The surgical decision for eventual combined approaches was made based on the level of planned sacrectomy, intrapelvicity of the tumor, eventual involvement of visceral/urogenital structures, lumbar spine, and iliac vessel encompassment. Patients with metastatic disease (other localizations than the sacrum) or without oncologic indication for sacrectomy were excluded. As secondary endpoints were complication rates, we focused on the rate of infection and wound complications requiring surgical revision. The postoperative ambulatory status and the bladder/bowel function were scored according to Biagini et al. [15]. Adequate pain control by analgesic therapy was performed in the postoperative period, and the patient undertook a physiotherapy course to maintain upright position and ambulation. Any adjuvant therapy was performed after a discussion with multidisciplinary themes and according to the guidelines for the individual underlying malignancy. During individual treatments, patients were followed up regularly as part of follow-up. The study aimed to analyze overall survival, disease-free survival, and any recurrence and metastasis after sacrectomy.

## 3. Results

This retrospective monocenter study included 19 patients who underwent surgery for sacral tumors. The demographic and clinical data are reported in Table 1. Of the 19 patients (females/males: 7/12), the mean age at the time of surgery was 48.9 years (range 17–81). Primary malignancy was present in 89.5% of patients (17 cases), while 2 patients were treated for sacral metastases due to thyroid carcinoma and a Brenner tumor. Three patients received a previous treatment (incisional biopsy, chemotherapy, radiotherapy); 84.2% of patients (16 cases) underwent preoperative selective arterial embolization (SAE). A total of 21.1% of the patients (four cases) underwent double access surgery, and in one case, anterior access only was performed; for all the other patients, posterior approaches were used. High sacral partial resection (above the S3 segment) was performed in four cases, (21.1%) of the patients, and low sacral resection (at or below the S3 segment) in the remaining cases. In 63.2% of all the patients, resection was performed completely with wide Enneking-appropriate surgical margins (R0 resection); in 36.8% (seven patients), the resection margin presented a microscopic tumor remnant (R1 resection, Enneking-inadequate). In five patients (26.3%), nerve root resection was performed to achieve wide margins or because it was incorporated into the primary tumor. In the case of high sacrectomy, the sacrifice of at least one S1 nerve root was performed and patients experienced initial postoperative weakness of the foot plantar flexion, but they were able to ambulate without external support within 6 months after surgery.

Postoperative radiation therapy was performed in five cases (26.3%) and four patients (21.1%) received postoperative chemotherapy. Local recurrence was observed in five patients (26.3%); two of them underwent reoperation with neurological sacrifice. Distant metastases occurred in four cases (21.1%).

The mean follow-up period was 49 months (range 13–127 months). We found that the length of stay (LOS) was related to the extent of sacral resection. The mean LOS was 16 days (5–42 days). Eight patients died during follow-up; the mean follow-up period for these patients was 51 months (range 22–85). Survival at 5 years after surgery was 73.7%.

The postoperative neurological status is reported in Table 2. All patients maintained their bowel function and their ambulatory status, even if one patient required external support after revision surgery for local recurrence; concerning bladder function, two patients were affected by limited continence after revision surgery for local recurrences.

**Figure 1:**
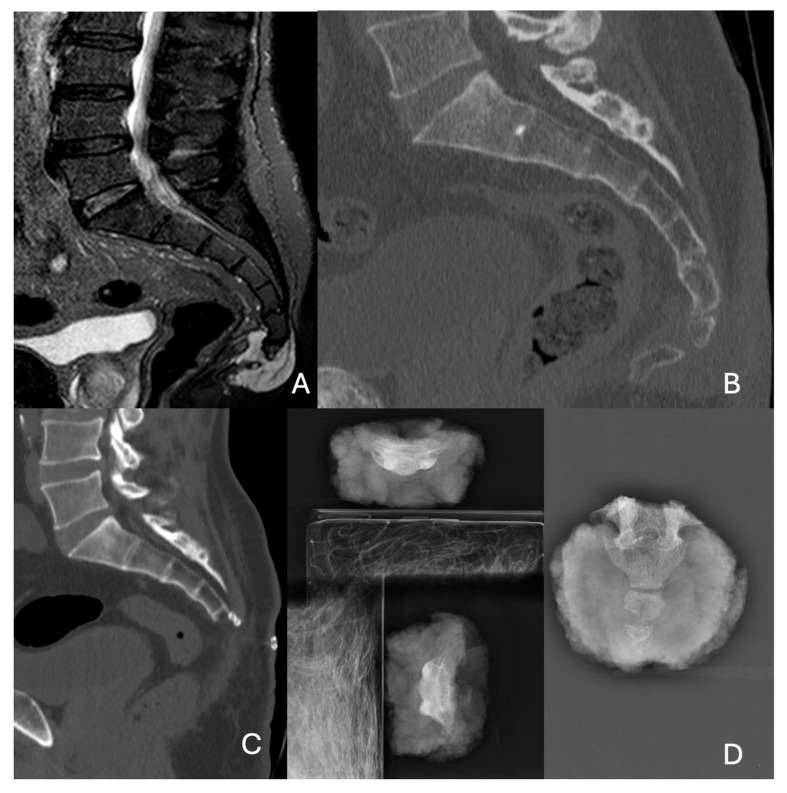
Preoperative MRI (**A**) and preoperative CT scan (**B**) of S4 chordoma. Postoperative CT scan after midline resection at S4 level (**C**). X-ray of the anatomical surgical specimen (**D**).

**Figure 2:**
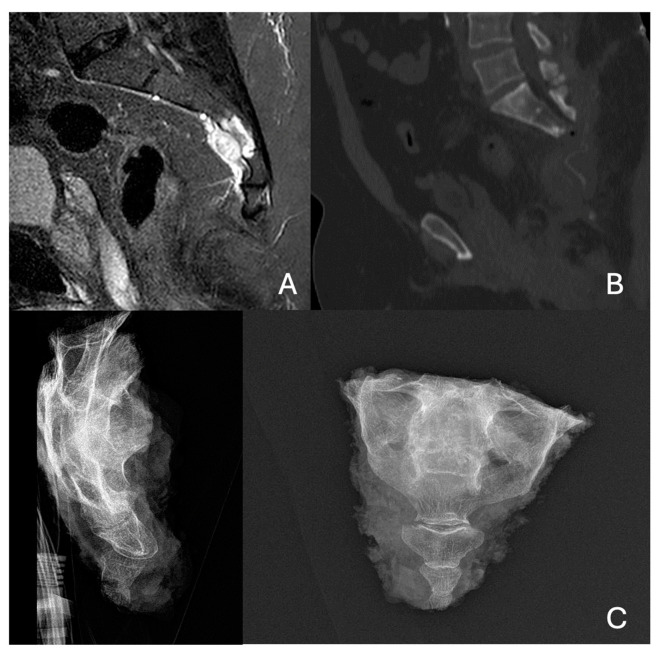
Preoperative MRI (**A**) and preoperative CT scan (**B**) of mid-sacral chordoma. X-ray of the anatomical surgical specimen (**C**).

Two cases of resection for sacral tumors are illustrated in Figure 1 and Figure 2.

### Complications

Postoperative complications occurred in 12 patients (63.2%). Of these complications, five were surgical wound dehiscence with delayed healing and one case required surgical debridement without revision. No mechanical complications occurred. Early minor complications occurred in seven patients: five urinary tract infections and two postoperative ileus resolved with pharmacological treatment.

## 4. Discussion

Sacrectomy represents a major challenge in the field of orthopedic oncology and neurosurgery. This article explores the latest evidence on sacrectomy, highlighting the various surgical techniques, outcomes, and prospects. Over the years, several surgical approaches to sacrectomy have been proposed and evaluated. The exclusive posterior approach for en bloc resection of the sacrum has been shown to be effective, as reported by Clarke et al. (2012), who observed good clinical results with a posterior approach in 36 patients [16]. McLoughlin et al. (2008) further confirmed that total en bloc resection through a single posterior intervention can improve outcomes compared with less radical approaches [17]. However, it is important to note that the combined antero-posterior approach, as discussed by Pu et al. (2021), may offer advantages in terms of complete resection and subsequent reconstruction [13]. The morbidity and mortality associated with sacrectomy are critical aspects that affect patients’ postoperative quality of life. Branco et al. (2022) conducted a systematic review on primary bone tumors treated with sacrectomy, revealing significant variability in complication and mortality rates [18]. Postoperative complication rates and mortality are influenced by the complexity of the surgery and the patient’s general condition. Weidlich et al. (2024) confirmed that oncologic and surgical outcomes after sacrectomy for primary malignancies and local recurrences are variable, with great care needed for postoperative management [19]. It is clear how the experience of the individual center and different surgeons can influence the type of surgical approach. Currently, authors in the literature agree on the use of single posterior access for the treatment of sacral lesions below the S2 segment (exceptions are lesions growing anteriorly within the pelvis). During this surgical approach, it is essential to achieve optimal detachment of presacral adhesions and adequate control of the osteotomy, which therefore requires a high level of experience of the surgeon performing the procedure [16,17,20]. The limitation of this posterior approach is the poor visibility of the structures present in the anterior portion of the pelvis and thus, if there are vascular lesions, poor control of bleeding phenomena. In our study, in 85% of the patients, a posterior-only approach was performed. In our study, lesions above S2 were also treated with posterior access. Double access was performed in a case where there was expansion of disease at the visceral level from Brenner tumor metastasis and a hemibasin resection with reconstruction with a 3D prosthesis. In this case, a total sacrectomy with translumbar amputation was performed and, consequently, a complex reconstruction was required. Three-dimensional printing technology was used to create a tailored implant that enhanced preoperative planning and provided better biomechanical fit. The implant design was biomechanically evaluated by personalized finite element analysis. The implant was characterized by a porous structure with a low modulus of elasticity and a high coefficient of friction to enhance the contact surface and distribute vertical forces, thereby ensuring sufficient biomechanical stability to obtain osseointegration.

In the first double-access surgery (Brenner tumor), hemicolectomy and right nephrectomy were also performed. In the single anterior access, curettage was performed for an osteoid osteoma located in the anterior part of S2. Compared to the literature, our data are quite similar to those of Varga’s group, who in their work used predominantly posterior approaches in 76% of cases [21]. Wang et al., as in our study, described a posterior approach in 80% of their patients undergoing total, subtotal, or hemisacrectomy [1]. Other authors, such as Weidlich, performed combined antero-posterior approaches in 67% of patients. This very discordant value from the rest of the authors and from the values in our study can be explained by the high rate of total and high sacrectomies (52%) and the presence of locally recurrent rectal carcinoma [19]. Among the various authors, Ozdemir et al. prefer a purely posterior approach only for tumors distal to S, using a combined approach for tumors above S2 [6]. The experience of our group also demonstrates how the single posterior approach (in 17 patients in our study), when possible, clearly reduces intraoperative time with an average time of 2 h and 30 min (1–4 h), reducing the infectious and intraoperative bleeding risk, especially when compared to the average 12 h with double access (10–14 h). Our primary endpoint is patient survival. Obviously, there are several factors that influence patient survival: first of all, there is the biology of the primary tumor or the presence of metastasis and the stage of the pathology (having infiltrated surrounding tissues or having manifested distant metastasis); of course, the response to adjuvant/neoadjuvant treatments is also an important factor to consider. Our study had a minimum one-year follow-up survival of 100% of the patients with a mean follow-up of 31 months (12 m–128 m), which is comparable to other studies in the literature with a follow-up of 33–53 months [22,23]. Predictive factors for long-term survival include wide-margin resection [5,7,8,9]. Factors influencing metastasis after sacrectomy surgery have not yet been elucidated in the literature. However, it is known that clean surgical margins (R0 resection) not only appear to reduce the risk of local recurrence but are critical in significantly improving overall survival [19,21]. Contrasting on this aspect are Wang’s group and Hulen’s group. The former group also found a significant difference in survival and a lower risk of local recurrence in patients (R0) [1]. In contrast, Hulen et al. reported no significant difference in survival and local recurrence in the R0 and R1 group of patients or in terms of preoperative and postoperative radiotherapy [24]. In our experience, we had six disease progressions; in four of these cases, intralesional surgery had been performed with local progression of disease. In the other two operations performed at wide margins, the first was a chordoma and manifested metastatic lung lesions 7 years later, and in the second case a chordoma patient presented with a femoral lesion 8 months later. Thus, our data are in agreement with the current literature that a wide-margin resection reduces the risk of local recurrence but does not give guarantees about the possibility of developing distant metastases. In patients with reduced operability, an alternative strategy has been described by Du et al. [5]. The authors use palliative curettage alone, showing rapid pain reduction with improved quality of life. Clearly, this approach has a decreased complication rate, but it also has very low survival rates, i.e., 41% and 22.5% [5]. In our case series, only two patients were older than 60 years, and thus age could not be identified as a significant influencing factor for survival. In contrast, other studies, such as that by Samson et al., have shown that higher age seems to have a tendency for increased recurrence in sacral chordomas [25]. Also, in sacral chordomas, Cheng et al. came to similar conclusions, as they showed that higher age and high sacral tumor location are associated with higher recurrence rates [26]. On the other hand, Varga et al. did not find an effect of high age on the recurrence rate, but on the overall survival of patients with sacral chordoma [21]. One of our primary end points was to evaluate the disease-free survival of patients treated with sacrectomy. The end point was to evaluate the rate of recurrence in view of the different malignancies with which the subjects examined were affected. Among our patients, we had six local recurrences, of which three had chordoma and, respectively, one had metastasis from thyroid cancer, one had metastasis from a Brenner tumor, and another had chondrosarcoma. The last three tumors exhibit intrinsically more aggressive behavior; so, the risk of recurrence was expected. From the documentation in the literature, we wondered about the risk of recurrence, particularly of chordoma; thus, we evaluated the different aspects described by other authors. Vargas et al. identified tumor size, especially in those patients who had undergone previous surgery on the same side, and the type of resection (wide-margin or intralesional) as influencing factors [21]. The patient’s age, on the other hand, was considered irrelevant by the group of George et al. [9]. Van Wulfften Palthe et al., in their study, were able to document that tumor size is associated with worse overall and recurrence-free survival [27]. In contrast, the group of Colangeli et al. identified an association between high resections and lower overall survival associated with a higher risk of recurrence [23]. No intraoperative complications such as death, hemorrhage, or hemorrhagic shock occurred in our study. The zero intraoperative complication rate may be due to the fact that all patients were meticulously planned in terms of optimal local staging (MRI, PET, and CT) to assess size and sacral/pelvic invasion and any adhesions. Nerve root resections were evaluated preoperatively and determined using imaging. In our study, we had surgical wound infections and sphincter changes as postoperative complications. It is described in the literature that most common postoperative complications after sacrectomy are surgical site infection, wound dehiscence, failure of mechanical instrumentation, secondary cerebrospinal fluid leakage, urinary disorders, and sensorimotor dysfunction/fecal incontinence. Sacrectomy sphincter-type alterations are an almost invariable consequence of oncologic sacral nerve resections in high sacrectomies. The most frequent complications are healing disorders and wound dehiscence, and infections are extremely frequent and described in all current studies [1,16,18,25]. In the literature, the rate of infections described after sacrectomy varies between 25% and 60% [1,18,22]. In the present study, we had infections in 35% of cases corresponding to the literature data. The morbidity and mortality associated with sacrectomy are critical aspects that affect patients’ postoperative quality of life. Branco et al. (2022) conducted a systematic review on primary bone tumors treated with sacrectomy, revealing significant variability in complication and mortality rates [18]. Postoperative complication rates and mortality are influenced by the complexity of the surgery and the patient’s general condition. Weidlich et al. (2024) confirmed that oncologic and surgical outcomes after sacrectomy for primary malignancies and local recurrences are variable, with great care needed for postoperative management [19].

## 5. Conclusions

Sacrectomy remains a complex procedure with significant challenges in terms of surgical and oncologic outcomes. The present study highlights both the successes and challenges of sacrectomy. While surgical techniques have shown the ability to achieve large resections, postoperative complications remain a significant concern. Predicting the functional prognosis of sacral primary tumors by classifying sacral resections according to the degree of nerve root sacrifice reiterates the need for appropriate preoperative planning. It is essential to continue to monitor patients long-term to effectively manage recurrence and metastasis, and to optimize treatment and postoperative management strategies. This study contributes to our understanding of sacrectomy, but further research is needed to refine surgical techniques, reduce complications, and improve long-term oncologic and functional outcomes.

## Figures and Tables

**Table 1:** Demographic and clinical data.

Variable	Category	Number of Patients (%)	Mean
Gender	Female	7 (36.8%)	
	Male	12 (63.2%)	
Age at surgery			48.9 years (range 17–81)
Charlson Comorbidity Index			3.7 (range 2–7)
Pain	Yes	19 (100%)	
	No	0	
Type of tumor	Chordoma	11 (57.9%)	
	Chondrosarcoma	2 (10.5%)	
	Spindle cell sarcoma	1 (5.3%)	
	Osteoid osteoma	1 (5.3%)	
	ABC	1 (5.3%)	
	Malignant myofibroblastic tumor of bone	1 (5.3%)	
	Metastases	2 (10.5%)	
Previous treatments	Yes	3 (1 incisional biopsy; 1 radiation therapy; 1 chemotherapy)	
	No	16	
Preoperative SAE	Yes	16 (84.2%)	
	No	3 (15.7%)	
Type of surgery	High sacral partial resection (above the S3 segment)	4 (21.1%)	
	Low sacral resection (at or below the S3 segment)	15 (78.9%)	
Surgical margin	R0	12 (63.2%)	
	R1	7 (36.8%)	
Surgical approach	Posterior	14 (73.7%)	
	Anterior	1 (5.3%)	
	Double (posterior + anterior)	4 (21.1%)	
Neurological sacrifice	Yes	5 (26.3%)	
	No	14 (73.7%)	
Adverse events	Postoperative	12 (63.2%)	
		5 wound dehiscence	
		7 minor complications	
Length of stay			15.7 days
Local recurrence	Yes	5 (26.3%)	
	No	14 (73.7%)	
Metastases/other localizations	Yes	4 (21.1%)	
	No	15 (78.9%)	
Follow-up			49.1 months (range 13–127)
Adjuvant treatments	RT	5 (26.3%)	
	CHT	4 (21.1%)	

**Table 2:** Postoperative ambulatory status and bladder/bowel functions, according to Biagini et al. [15].

Function	Score	Postoperative Status	Number of Patients
Motor	0	Normal or mild deficit; does not require external support	18
	1	Deficit requiring help of external support	1 (Revision for LR)
	2	Deficits that make walking impossible	0
Bladder	0	Normal	17
	1	Feels stimulus to micturate and has limited continence at varying times	2 (Revision for LR)
	2	Does not feel stimulus to micturate or is completely incontinent	0
Bowel	0	Normal	19
	1	Feels stimulus to defecate and is incontinent under stress	0
	2	Does not feel stimulus to defecate or is completely incontinent	0

## Data Availability

Data supporting reported results can be found in the digital archive of Istituto Ortopedico Rizzoli.
